# A non-inferiority randomized phase III trial of standard immunotherapy by checkpoint inhibitors *vs.* reduced dose intensity in responding patients with metastatic cancer: the MOIO protocol study

**DOI:** 10.1186/s12885-023-10881-8

**Published:** 2023-05-02

**Authors:** Gwenaelle Gravis, Patricia Marino, Daniel Olive, Frederique Penault-LLorca, Jean-Pierre Delord, Clotilde Simon, Assia Lamrani-Ghaouti, Renaud Sabatier, Joseph Ciccolini, Jean-Marie Boher

**Affiliations:** 1 Department of Medical Oncology, Institut Paoli-Calmettes, Aix-Marseille University, CRCM,, Marseille, France; 2 Institut Paoli-Calmettes SESSTIM UMR 1252, INSERM, IRD, Aix Marseille University, Marseille, France; 3 Institut Paoli Calmettes, Aix Marseille Université, CRCM, Inserm U1068, Marseille, France; 4 Centre Jean Perrin, Université Clermont Auvergne, INSERM, U1240 Imagerie Moléculaire Et Stratégies Théranostiques, Clermont Ferrand, France; 5 Institut Claudius Regaud IUCT Oncopole, Toulouse, France; 6 Unicancer R&D, Paris, France; 7 AP-HM Inserm U1068, Marseille, France; 8 Department of Biostatistics and Methodology, Institut Paoli Calmettes, and Aix-Marseille University, Unité Mixte de Recherche S1252, Institut de Recherche Pour Le Développement, 13009 Marseille, France

**Keywords:** Metastatic cancer, Immunotherapy, Checkpoint inhibitors, Health Economics, Quality of life

## Abstract

**Background:**

Immunotherapy (IO) has become a standard of care for treating various types of metastatic cancers and has significantly improved clinical outcome. With the exception of metastatic melanoma in complete response for which treatment can be stopped at 6 months, these treatments are currently administered until either disease progression for some IO, 2 years for others, or unacceptable toxicity. However, a growing number of studies are reporting maintenance of response despite discontinuation of therapy. There is currently no evidence of a dose effect of IO in pharmacokinetic studies. Maintaining efficacy despite a reduction in treatment intensity by decreasing the frequency of administration in patients with highly selected metastatic cancer, is the hypothesis evaluated in the MOIO study.

**Method/design:**

This non-inferiority, randomized phase III study aims to compare the standard regimen to a 3 monthly regimen of variousIO drugs in adult patients with metastatic cancer in partial (PR) or complete response (CR) after 6 months of standard IO dosing (except melanoma in CR). This is a French national study conducted in 36 centers. The main objective is to demonstrate that the efficacy of a three-monthly administration is not unacceptably less efficacious than a standard administration. Secondary objectives are cost-effectiveness, quality of life (QOL), anxiety, fear of relapse, response rate, overall survival and toxicity.

After 6 months of standard IO, patients with partial or complete response will be randomized 1:1 between standard IO or a reduced intensity dose of IO, administered every 3 months. The randomization will be stratified on therapy line,, tumor type, IO type and response status. The primary endpoint is the hazard ratio of progression-free survival. With a planned study duration of 6 years, including 36 months enrolment time, 646 patients are planned to demonstrate with a statistical level of evidence of 5% that the reduced IO regimen is non-inferior to the standard IO regimen, with a relative non-inferiority margin set at 1.3.

**Discussion:**

Should the hypothesis of non-inferiority with an IO reduced dose intensity be validated, alternate scheduling could preserve efficacy while being cost-effective and allowing a reduction of the toxicity, with an increase in patient’s QOL.

**Trial registration:**

NCT05078047.

**Supplementary Information:**

The online version contains supplementary material available at 10.1186/s12885-023-10881-8.

## Introduction

The number of approved immune-oncological agents (IO) such as PD-1/PD-L1 and CTLA-4 inhibitors is increasing. Their rhythm and duration of treatment are recommended until either disease progression for some IO, 2 years for other or unacceptable toxicity. However, there are still some unresolved questions about the administration schemes of IO.

First, the optimal duration of immune checkpoint inhibitors (ICIs) is currently unknown. Complete response (CR) before therapy discontinuation is shown to be a positive factor for a prolonged response as shown in the KEYNOTE-001 & 006 trials [1, 2]. To date it is not clear whether continuous immunotherapy treatment should be given to responding patients until disease progression, since stable responses and long survivals have also been reported in patients in whom immunotherapy had been discontinued months before, with an actual survival plateau observed in melanoma, non-small cell lung cancer (NSCLC) and renal cell cancer [3, 4]. This durability of the response is currently explained by the theoretical rationale that anti–PD-L1 therapy can generate a mono or polyclonal and memory adaptive T-cells anti-tumor immunity, either CD45 or CD86 that is able to control the heterogeneity of the disease and to reset the tumor-host immune interaction toward cancer rejection [5].

Second, the minimal duration of ICIs is also currently unknown. The Evidence from current clinical trials in different tumor types indicates that most of the responses are generally occurring early, with a median time of 2–4 months. In patients with CR, the risk of progression was significantly higher in those treated for < 6 months compared with those treated for > 6 months [6]. CheckMate153 that compared continuous *versus* 1-year immunotherapy in previously treated advanced NSCLC in partial or complete response, or in stable disease, suggested that continuing nivolumab beyond 1 year improved outcomes [7].

The frequency of administration has been arbitrary defined by clinical trials. The optimal dose of IO remains unknown. No major dose-dependent effect of anti-PD-1 has been observed, and whether the frequency of infusion of IO could improve response or maintain efficacy is unclear. Moreover, phase I studies have shown that saturation of the target (PD-1 or PD-L1) can persist far beyond the serum half-life of the IO and 3-monthly infusions of an anti-PD-1 antibody could potentially generate the same level of activity as infusions administered every 2 weeks [8]. In silico modelling studies have suggested that alternate scheduling with IO would not compromise treatment efficacy. Indeed, prolonged half-lives of IO drugs, time-varying clearance plus plasma concentrations far above the threshold associated with maximal target-engagement, suggest that the rhythm of administration of IO could be slowed down [9–12].

In a context were over-treatment with ICIs may be toxic, inefficient [13] and cost-ineffective [14], a prospective analysis of progression-free survival and overall survival between continuous treatment *versus* reduction treatment without discontinuation is an alternative that need to be evaluated. This therapeutic alternative may provide less drug-related SAEs, with better quality of life and reduced treatment costs.

MOIO is a randomized phase III study comparing the standard administration of IO *versus* the same IO administered every 3 months in patients with metastatic cancer in partial or complete response after 6 months of approved standard IO (except melanoma in complete response).

## Methods & design

### Study design

Sponsored by the French national network of anticancer centers (UNICANCER), MOIO is a national, multicenter, randomized, controlled, open label Phase 3 trial comparing a reduced dose intensity of IO *versus* approved standard IO regimen in patients with metastatic oncologic tumor in complete or partial response after 6 months of treatment with standard IO. Study protocol has been approved by the Institutional Review Board (IRB) of UNICANCER.

#### Study objectives

The primary objective of the trial is to demonstrate the non-inferiority in term of PFS of administration of reduced dose intensity of IO *versus* standard IO for patients in response after 6 months of standard IO.

Secondary objectives are to evaluate:

- Cost-effectiveness,
- The efficacy in terms of:
  - Immune progression-free survival using iRECIST,
  - Objective response rate at 12 and 24 months post-randomization,
  - Overall survival,
  - Duration of response at 12 months post-randomization,
-  Quality of life (self-reported EORTC QLQ-C30 and EQ-5D-5L questionnaires),
-  Anxiety and fear of relapse using specific questionnaires,
-  Safety profile.

#### Primary endpoint

The primary outcome is progression-free survival (PFS) calculated from the date of randomization to the date of first progression or death from any cause, whichever occurs first. Progression will be determined locally by the investigator through the use of RECIST v1.1 in case of lesions identified at baseline. For patients without any evidence of disease at inclusion, the progression will be defined as an appearance of a new lesion (measurable or not measurable). Patients who have not progressed or died at the time of analysis will be censored at the time of the latest date of RECIST assessment.

In case of initiation of subsequent anticancer therapy before disease progression or death, patients will be censored on the date of first initiation of subsequent anticancer therapy.

#### Secondary endpoints

The secondary outcomes are:

- Incremental cost-effectiveness ratio (ICER) expressed as a cost per quality-adjusted life year (QALY) gained at 36 months.
- Efficacy
  - Immune progression-free survival (iPFS) calculated from the date of randomization to the date of disease progression or death from to any cause, whichever occurs first. Immune progression will be determined locally by the investigator according to iRECIST v1.1 in case of lesions identified at baseline.
  - Objective response rate (ORR) defined as the percentage of patients with a confirmed complete response (CR) or partial response (PR) at 12 and 24 months post-randomization considering patients who switch from study treatment to any other cancer treatment within 12 and 24 months post-randomization as failures.
  - Overall survival (OS) calculated from the date of randomization to the date of death from any cause.
  - Duration of response (DoR) defined as the time from randomization to first documented disease progression or death, whichever occurs first.
- Quality of life mean score of self-reported EORTC QLQ-C30 and EQ-5D-5L questionnaires at inclusion visit (pre-randomization), 3, 6, 9, 12, 15, 18, 24 and 36 months post-randomization.
- Evaluation of anxiety and fear of recurrence scores using specific questionnaires (HADS and Fear of Cancer Recurrence Inventory, Short Form) at 3, 6, 9, 12, 15, 18, 24 and 36 months post-randomization.
- Number, frequency and severity of adverse events according to CTCAE v5.0 at 12 months and 3 years post-randomization.

Ancillary studies of pharmacokinetics, immuno-monitoring and ctDNA will be conducted to determine biomarkers (at the immune and genomic levels) that can identify patients that would benefit from dose reduction.

## Study population

Prior to enrollment, all participants will be screened to check their inclusion and exclusion criteria (Table 1).

**Table 1:** Eligibility criteria for the study population

Inclusion criteria	Exclusion criteria
1. Patient must have signed a written informed consent form prior to any trial specific procedures 2. Patient aged ≥ 18 years old 3. Initial metastatic disease histologically confirmed including: lung cancer, renal cell cancer, head and neck cancer, bladder cancer, triple negative breast cancer, Merkel cancer, hepatocellular carcinoma, melanoma, colorectal carcinoma with microsatellite instability [MSI], or esophageal squamous cell carcinoma 4. Patients in partial or complete response after 6 months of standard immunotherapy (whatever the line of therapy) according to the RECIST criteria (confirmed by local radiological assessment). For metastatic melanoma only patients in partial response 5. Eligible to maintain the same standard IO treatment 6. Patient with ECOG performance status ≤ 1 7. Patients with brain metastases are allowed, provided they are stable according to the following definitions: treated with surgery or stereotactic radiosurgery and without evidence of progression prior to randomization and have no evidence of new or enlarging brain metastases 8. Patients treated by IO previously combined with chemotherapy are allowed 9. Patients with TKI-IO or pemetrexed-IO or bevacizumab-IO are allowed 10. Evidence of post-menopausal status, negative urinary, or serum pregnancy test for female pre-menopausal patients 11. Both sexually active women of childbearing potential and males (and their female partners) patients must agree to use adequate contraception method for the duration of the study treatment and after completing treatment according to the most recent version of the IO SmPC 12. Patient is willing and able to comply with the protocol for the duration of the trial including undergoing treatment and scheduled visits, and examinations including follow-up 13. Patient must be affiliated to a Social Security System	1. Metastatic melanoma in complete response 2. Metastatic renal cell carcinoma with IMDC favourable-risk treated TKI/IO combination 3. Hematologic malignancies (leukaemia, myeloma, lymphoma…) 4. Active infection requiring systemic therapy 5. Patients enrolled in another therapeutic study within 30 days before the inclusion in and during MOIO study 6. Patient unable to comply with study obligations for geographic, social, or physical reasons, or who is unable to understand the purpose and procedures of the study 7. Person deprived of their liberty or under protective custody or guardianship

### Inclusion criteria

Eligible patients should have a histologically confirmed metastatic disease (lung cancer, renal cell cancer, head and neck cancer, bladder cancer, triple negative breast cancer, Merkel cancer, hepatocellular carcinoma, melanoma, colorectal carcinoma with microsatellite instability (MSI) or esophageal squamous cell carcinoma). Patients with brain metastases are allowed, provided they are stable (treated with surgery or stereotactic radiosurgery and without evidence of progression prior to randomization and have no evidence of new or enlarging brain metastases). Patients should be in partial or complete response after 6 months of standard immunotherapy (whatever the line of therapy). For metastatic melanoma, only patients in partial response will be eligible. Patients treated in the first 6 months by IO alone or in combination with other IO, chemotherapy or tyrosine kinase inhibitor (TKI) are also allowed. Patient must be 18 years or older, have a good general health status (ECOG ≤ 1), eligible to maintain the same standard IO treatment, and provide a signed informed consent form before study entry.

### Exclusion criteria

Patients are not eligible to participate in the trial if they have metastatic melanoma in complete response, metastatic renal cell carcinoma with IMDC favorable-risk treated TKI/IO combination, or hematologic malignancies (leukemia, myeloma, lymphoma…).

## Randomization

Eligible patients will be randomized 1:1 to:

1. *Experimental arm*: Reduced dose intensity of IO

IO will be administered every 3 months (at the same dose levels) until disease progression, unacceptable toxicity, death, patient’s choice or investigator’s decision.

2. *Control arm*: Standard IO

Continuation of IO at the same dose levels and rhythmicity until disease progression, unacceptable toxicity, death, patient’s choice or investigator’s decision.

The randomization will be stratified by:

- Response (CR vs PR),
- Metastatic tumor type (lung cancer, renal cell cancer, head and neck cancer, bladder cancer, triple negative breast cancer, Merkel cancer, hepatocellular carcinoma, melanoma, colorectal carcinoma with microsatellite instability [MSI], or esophageal squamous cell carcinoma).
- IO type (anti-PD-1 vs anti-PD-L1),
- Treatment line (first line vs others).

**Fig. 1:**
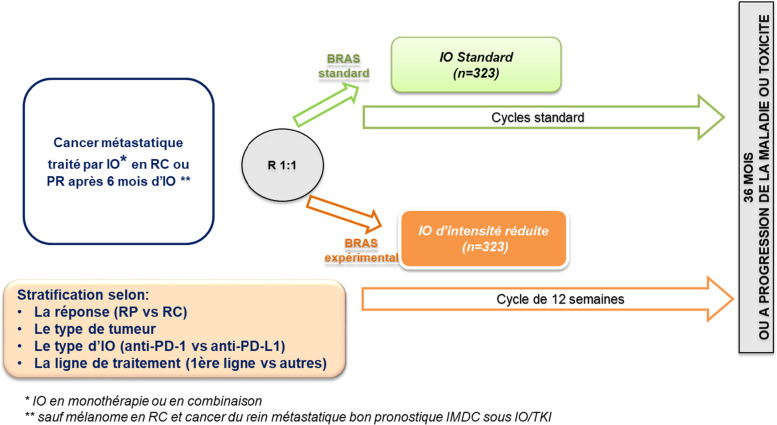
Trial scheme of the MOIO clinical trial

Trial design is shown in Fig. 1.

## Treatment plan

### Therapeutic regimens

Patient treatment will consist of immunotherapy with PD-1 or PD-L1 antagonists to be used in monotherapy or in combination (with other immunotherapy [ipilimumab] or chemotherapy or continuous combination with pemetrexed, bevacizumab or TKI), within their marketed indication and according to most recent version of the Summary of Product Characteristics (SmPC) of each product (Table 2).

**Table 2:**
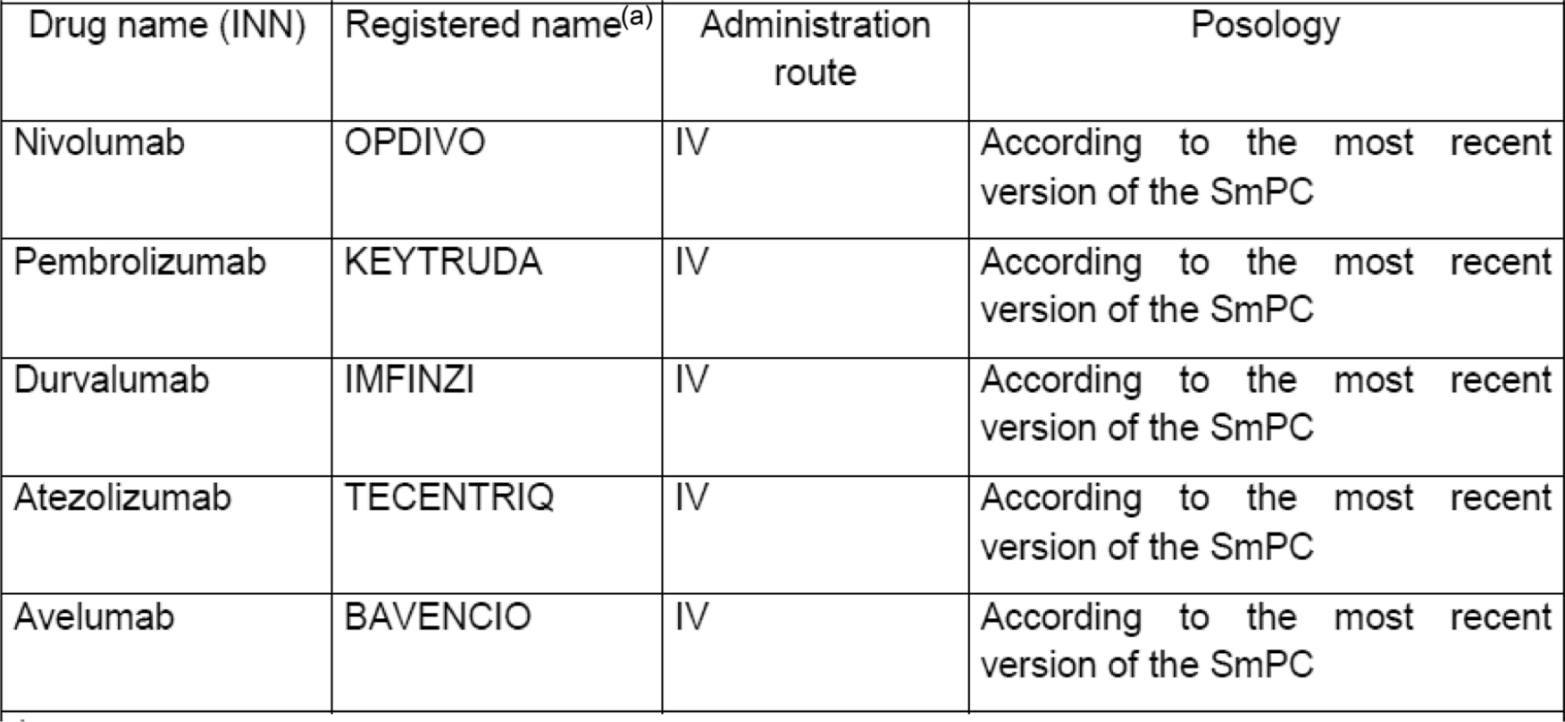
Product name and administration

*INN* International non-proprietary name, *SmPC* Summary of product characteristics

^a^When any generic drug can be used indicate only the INN Name. The choice of the registered name or brand name used in the trial is at the investigation center discretion

Treatment should be administered and adjusted and toxicities managed in both arms according to the most recent version of the IO SmPC recommendations and local practice.

### Duration of treatment

Treatment duration will be 36 months maximum or until disease progression, unacceptable toxicity, death, patient’s choice or investigator’s decision.

### Visit schedule and assessments (Supplementary Table 1)

MOIO patients treated with IO in both arms will have a follow-up during 36 months post-randomization. This first visit take place immediately after randomization. During the treatment and follow up periods, patients in both arms will have a trial visit every 3 months (± 7 days). However, depending on treatment tolerance, additional visits may be scheduled as per standard of care. All examinations revealing a toxicity related to IO treatment must be periodically repeated until toxicity disappearance (or until it is deemed irreversible) as per standard of care. The last visit corresponding to the end of MOIO study will be done 36 months post randomization.

### Adverse events

All AE, irrespective of seriousness, will be collected from the day of randomization to the follow-up visit/end of trial visit at the time points specified in the trial visits schedule.

Each AE associated with treatment in both arms will be classified using the NCI-CTCAE v5.0 classification.

## Statistical analysis

### Sample Size calculation

Given a HR of 1.3 as the non-inferiority margin, 498 progressions or deaths are necessary to establish the non-inferiority in PFS of the experimental arm *versus* the control with 5% level of significance and 90% power assuming no difference between arms (HR = 1).

Assuming 22-month median PFS in both arms, 36-month accrual period, 72-month study duration and a 5% lost to follow-up rate, a total of 646 patients is planned.

The final analysis is scheduled 3 years after the last patient randomization.

### Statistical analyses

The assessment of efficacy will be based primarily on a per-protocol (PP) population consisting of all randomized patients, except those who did not adhere to the study treatment for reasons of personal convenience, which could make the treatment groups appear similar. Other analyses will be based on a modified intention-to-treat population (mITT) consisting of all randomized patients, except patients who were deemed ineligible after randomization or patients who never started treatment. Prior to analysis, exclusion from PP and mITT populations will be reviewed by an independent data monitoring committee. Unless otherwise specified, the parameters will be estimated with 95% confidence intervals and missing data will not be replaced.

#### Analysis of the primary outcome

The primary analysis will seek to evidence non-inferiority in PFS of the experimental arm *versus* the active control arm on the per protocol population.

The PFS data will be summarized in each treatment arm using Kaplan–Meier methods. The analysis will be based on a Cox proportional hazards model including terms for the randomized arm, the tumor type, the IO type (anti-PD-1 vs anti-PD-L1), the therapy line (1st line vs others) and the response status 6 months after initiation of standard IO.

Non-inferiority in PFS will be evaluated on the per protocol set. Cox’s regression analyses including terms for randomization factors will be used to estimate the hazard ratio for treatment arm (reduced IO vs standard IO) with 90% confidence interval. Non-inferiority will be declared if the upper bound of the 90% bilateral confidence interval is lower than the predefined non-inferiority margin. In case of non-adherence to the study treatment, a secondary analysis will be conducted on the modified ITT set. Following CPMP guidelines, non-inferiority will be claimed if both analyses lead to similar conclusions.

#### Interim analysis

In order to minimize the number of patient exposed to an inferior treatment, an independent data monitoring committee will review the efficacy data to stop the trial early for lack of efficacy (harm or futility reasons). Two interim analyses are planned when 25% (*n* = 125) and 50% (*n* = 249) of the total planned number of events (*n* = 498) occurs. At each interim time, a simple and intuitive approach will recommend to stop the trial if the one-sided p-value for testing the hypothesis HR = 1 *versus* the alternative HR > 1 is less than 0.0192 (*p*-value associated to an observed HR = 1.3 at mid-trial) [15].

#### Analysis of secondary outcomes

- Cost-effectiveness

The endpoint of the economic analysis will be incremental cost-effectiveness ratio (ICER) which defines the cost per quality-adjusted life years (QALYs). QALYs will be estimated from OS and Progression-free survival PFS by weighting each mean survival time by a utility value (ranging from 0 to 1) derived from the EQ-5D-5L questionnaire. Cost-effectiveness acceptability curves, which give an estimate of the probability that the reduced-dose strategy is cost-effective given various values of society’s willingness to pay, will be computed. Unadjusted mean differences in costs and QALYs between the treatment groups will be reported with associated 95% confidence intervals. Tests used for comparisons will be chosen depending on the normality or not of cost data distribution. Comparison of QALYs between groups will be computed by using bilateral logrank tests. To capture longer-term expected costs, outcomes and cost-effectiveness, in a secondary analysis a simulation model will be developed over a 10-year horizon using a probabilistic Markov model. The transition probabilities between patient’s health states (toxicity, stable disease, progression, death) will be obtained both from the study and from the available literature at the date of analysis.

- iPFS, DoR and ORR will be analyzed on the PP and the mITT population. Efficacy time-to-event outcomes will be censored on the date of last known follow up visit. In case of initiation of subsequent anticancer therapy before disease progression or death, individual iPFS and DoR data will be censored on the date of first initiation of subsequent anticancer therapy.
- ORR at 12 and 24 months post-randomization are defined as the percentage of patients with a confirmed CR or PR by considering patients who switch from study treatment to any other cancer therapy within 12 and 24 months post-randomization as treatment failures.
- Time-to-event outcomes will be summarized using Kaplan–Meier methods. Regression models with terms for the randomized arm, the tumor type, the IO type (anti-PD-1 vs anti-PD-L1), the therapy line (1st line vs others) and the response status 6 months after initiation of standard IO will be used to estimate the treatment effect (reduced IO vs standard IO) on secondary efficacy endpoints. Hazard ratios for iPFS, DoR and OS and odds ratio for ORRs will be estimated using Cox’s or logistic regression modelling, respectively.
- Analyses of self-reported questionnaires will be based on the mITT population set. Actual values and changes from pre-randomization visit in EORTC QLQ-C30, EQ-5D-5L, anxiety (HospitalAnxiety and Depression Scale) and fear of relapse. (Fear of Cancer Recurrence inventory short Form) specific questionnaires will be tabulated by treatment group using standard descriptive statistics. Differences in changes from pre-randomization visit between treatment arms will be estimated at specific time visit using robust linear regression methods for longitudinal data.

## Data management and monitoring

An independent data monitoring committee (IDMC) with expertise and experience in immunotherapy,and without direct involvement in the conduct of the trial, will be set up specifically to guarantee:

- Effective protection of patients.
- Insure the ethical conduct of the trial.
- Benefit/risk ratio of the trial.
- Ensure the independent review of the scientific results during the trial and at the end of the trial.

The IDMC will be composed of at least two oncologists, a health economist and a statistician.

In order to minimize the number of patient exposed to an inferior treatment, the IDMC will review the efficacy data to stop the trial early for lack of efficacy (harm or futility reasons).

Two interim analyses will be planned and presented to the IDMC:

- First analysis when 125 events are observed.
- Second analysis when 249 events are observed.

The IDMC may recommend the early termination of the trial if one of the following conditions is met:

- The results of the interim analysis clearly show that the one-sided P-value for testing the hypothesis HR = 1 versus the alternative HR > 1 is less than 0.0192 (*p* value associated to an observed HR = 1.3 at mid-trial);
- An unacceptable toxicity.

Database management will be provided by an electronic case report form (eCRF) developed using the CSOnline module of Ennov Clinical® software. Patients’ individual data are strictly confidential and will be available only to investigators and some defined authorized persons.

## Discussion

For approved immunotherapies, such as PD-1/PD-L1 inhibitors and anti-CTLA-4 agents, treatments are administered until disease progression or unacceptable toxicity, with the exception of metastatic melanoma in complete response for which treatment can be stopped at 6 months. However, the optimal dose, frequency and duration of these therapies are currently unknown and a growing number of studies are reporting maintenance of response despite discontinuation of therapy or extension of IO dose intervals [16, 17]. The PRIMS trial, a phase II in metastatic renal cell cancer presented at ESMO meeting 2021, has shown that extending treatment intervals of immunotherapy did not compromise efficacy and reduced toxicity [16]. A Phase II study in Merckel and metastatic melanoma explored reduced frequency dosing of anti-PD1 with significant efficacy and reduced logistical and financial burden [17].

### The interval of treatment

One of the challenges in the field of oncology is knowing how and when to de-escalate immunotherapy and to safely identify patient candidate to de-escalation. MOIO is a pragmatic and strategic study that compares for the first time in a randomized phase III study standard IO administration to the same agent administered every three months in patients with metastatic cancer in in partial or complete response after 6 months of IO (excluding melanoma patients in complete response).

We choose a non-inferiority study because it is the best way to demonstrate that de-escalation is not unacceptably less effective than standard IO scheduling. The population is highly selected and is limited to patients who are in partial or complete response after 6 months of standard IO alone or in combination with other treatments (TKI or chemotherapy).

Cost-effectiveness studies have shown contradictory results about efficiency of checkpoint inhibitors in various type of cancer [14, 18–20]. The possibility of de-escalating cancer treatments by reducing the duration, frequency, dose or number of therapies administered can be seen as an efficient way to optimize patient outcomes efficiently [21]. With the escalating price of cancer drugs [22] and healthcare expenses largely attributable to expensive drugs, the cost of treatment is an important consideration in clinical management decisions [23, 24] and increasing treatment access especially in low-income countries [25]. The cost-effectiveness analysis is therefore an important secondary objective in the MOIO study. While available studies evaluating the efficiency of IO focus on specific localizations, the originality of the MOIO study is to analyze a de-escalation strategy and its economic impact in a multi-disease context.

Beyond the cost-effectiveness of de-escalation strategies, quality of life and patients’ wellbeing is an important issue for cancer treatment decisions. Chronic administration of IO generates a significant burden for patients due to the risk of dose-dependent adverse effects, entailing multiple clinic visits and the risk of chronic, life changing and sometimes life-threatening immune mediated toxicities [26, 27]. Optimal dosing of these agents is therefore especially important to maintain patient’s quality of life while maintaining good clinical outcome. Consequently, a formal prospective QOL assessment is a secondary objective of the MOIO study. Increasing quality of life and reducing toxicity may have positive consequences on treatment compliance in the longer term.

Fear of disease recurrence may be a barrier to de-escalation clinical trials. This is one of the reasons why a reduced frequency of administration was preferred to treatment discontinuation. However, in the context of little available prospective data on efficacy, patients and physicians may be afraid to decrease drug dose intensity for fear of tumor progression. In particular, a risk is that patients think they are being undertreated and that the treatment choices are guided by financial rather than medical considerations. It is also established that fear of cancer recurrence is associated with impaired mental quality of life [28, 29]. Thus, we decided, using validated tools, to also assess recurrence of distress and anxiety using patient reported outcomes in the MOIO study.

## Conclusion

Many questions remain unanswered about the dose, frequency and duration of administration of immunotherapy by immune check -point inhibitors, particularly in this very sensitive population with a very good response. De-escalation strategies have multiple potential benefits, including reduced toxicity, improved quality of life, improved cost-effectiveness, and better compliance with therapy while maintaining good clinical outcomes. If the non-inferiority hypothesis is demonstrated in the French MOIO study, the use of reduced dose of IO could be practice changing and become the new standard of care for very good responder’s patients.

## Supplementary Information

**Additional file 1:** **Supplementary Table 1.** Schedule of visits.

## Data Availability

Not applicable.

## Abbrevations

CAGR: Compound annual growth rate
AE: Adverse event
CR: Complete response
CTCAE: Common terminology criteria for adverse events
DoR: Duration of response
ECOG: Eastern cooperative oncology group
eCRF: Electronic case report form
EORTC: European organization for research and treatment of cancer
HADS: Hospital Anxiety and Depression Scale
HR: Hazard ratio
ICER: Incremental Cost-Effectiveness Ratio
ICIs: Immune checkpoint inhibitors
IDMC: Independent data monitoring committee
IO: Immunotherapy
IRB: Institutional Review Board
iPFS: Immune progression-free survival
iRECIST: Immune Response evaluation criteria in solid tumours
ITT: Intent-to-treat
mITT: Modified intent-to-treat population
ORR: Overall response rate
OS: Overall survival
PD: Progressive disease
PD-1: Programmed cell-death-1
PD-L1: Programmed cell death ligand 1
PP: Per protocol
PR: Partial response
PFS: Progression-free survival
QALY: Quality-adjusted life years
QLQ: Quality of Life Questionnaire
QOL: Quality of life
RECIST: Response evaluation criteria in solid tumours
SAE: Serious adverse event
SD: Stable disease
SmPC: Summary of product characteristics
TKI: Tyrosine Kinase Inhibitor
