# Supplementary figures and images for: A non-inferiority randomized phase III trial of standard immunotherapy by checkpoint inhibitors vs. reduced dose intensity in responding patients with metastatic cancer: the MOIO protocol study
Source: BMC Cancer. 2023 May 2;23:393. doi: 10.1186/s12885-023-10881-8 (PMC10155443; doi:10.1186/s12885-023-10881-8)

**Supplementary Table 1: Schedule of visits**


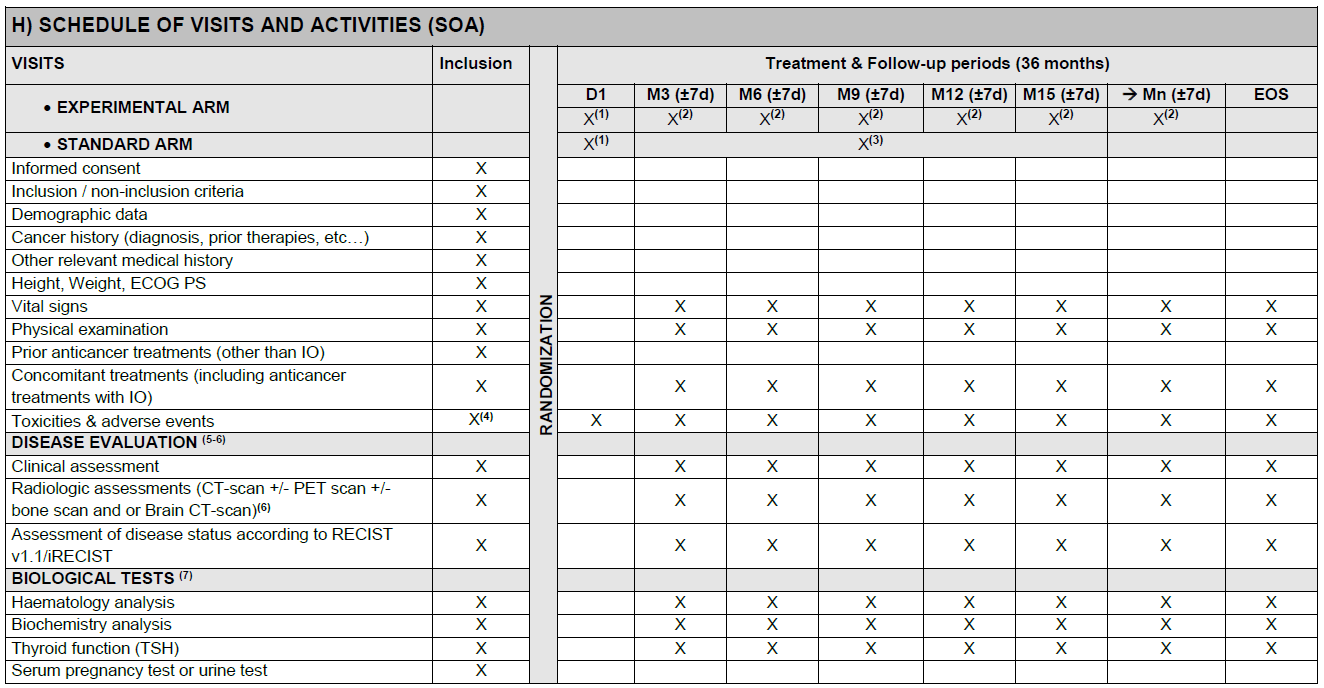


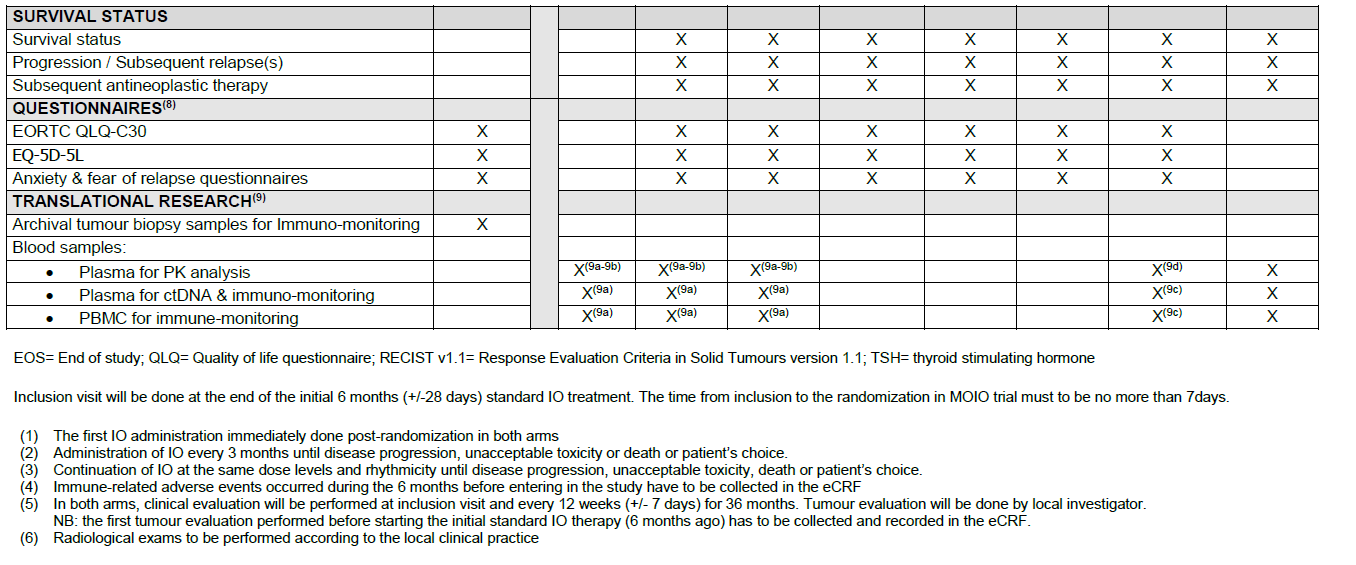

Supplement: Supplementary file 1 — Additional file 1: Supplementary Table 1. Schedule of visits. [file 12885_2023_10881_MOESM1_ESM.docx]
